# Sex and age are associated with host immune responses and multi-organ dysfunction in hospitalized patients with COVID-19

**DOI:** 10.3389/fpubh.2026.1936165

**Published:** 2026-08-31

**Authors:** Guochao Zhang, Yingying Zhao, Xiaoxia Chang, Qi Gao, Yunchao Zhang, Yanjun Lai

**Affiliations:** 1 Department of Clinical Laboratory, Ninth Hospital of Xi’an, Xi’an, Shaanxi, China; 2 Department of Pathology, Shanxi University of Medicine, Fenyang, Shanxi, China

**Keywords:** age, COVID-19, immune response, multi-organ dysfunction, sex

## Abstract

**Objectives:**

Sex- and age-related disparities are independent risk factors for poor COVID-19 outcomes, but underlying mechanisms remain unclear.

**Methods:**

This retrospective cohort study included 395 hospitalized COVID-19 patients (December 2022–March 2023). Biomarkers of multi-organ injury and immune responses were analyzed to evaluate the impact of sex and age on disease progression and clinical outcomes. Normality was assessed with the Shapiro–Wilk test; Student’s *t* or Mann–Whitney *U* tests were used accordingly, with Benjamini-Hochberg false discovery rate correction across 45 biomarkers. Multivariable logistic regression was used to adjust for sex, age, co-infection, comorbidities, and symptom onset to discharge time.

**Results:**

Among 395 patients (68% mild, 32% severe), males accounted for 76% of severe cases and patients aged ≥75 years for 79%. After false discovery rate correction (*q* < 0.05), 37 of 45 biomarkers differed by severity, 24 by sex, and 22 by age. Male patients had higher inflammatory, coagulation, cardiac, hepatic, and renal injury markers and lower lymphocyte, total protein, and albumin levels. Although not statistically significant, T, B, or NK cell counts showed decreasing trends in males. Older adult male patients displayed the most pronounced inflammatory responses, coagulation abnormalities, multi-organ injury, lymphopenia, and reduced nutritional reserves. In multivariable logistic regression, male sex (OR 1.925, 95% CI 1.063–3.484, *p* = 0.031) and age ≥75 years (OR 5.164, 95% CI 2.733–9.756, *p* < 0.001) were associated with severe COVID-19; the male sex and age association persisted after excluding co-infections and in patients with shorter onset-to-discharge durations.

**Conclusion:**

Sex and age differences are closely associated with COVID-19 severity, likely due to multi-organ dysfunction and immunosuppression following SARS-CoV-2 infection.

## 1 Introduction

COVID-19 is a highly contagious disease caused by severe acute respiratory syndrome coronavirus 2 (SARS-CoV-2). As of November 11, 2025, nearly 779 million individuals worldwide have been diagnosed with COVID-19, resulting in more than 7.1 million deaths. Among the deceased, older men aged ≥65 years account for a substantial proportion (1, 2). As early as March 2020, epidemiological reports indicated marked variations in infection rates and disease severity of COVID-19 (3). Several risk factors, including male, advanced age, and comorbidities—such as obesity, diabetes, hypertension, co-infections, and cardiovascular diseases—have been identified as independent predictors of severe disease, increased mortality, and poor prognosis (4–6). Clinical outcome analyses consistently demonstrate that men experience more severe disease and higher mortality rates than women (7, 8). Moreover, one large-scale study reported that men exhibited a higher sex-specific risk than women across three waves of the pandemic, a finding that could not be attributed to detection bias (9). Consistently, male patients remained at higher risk for severe disease and mortality even after adjustment for comorbidities and socioeconomic factors (9). The association between advanced age and increased COVID-19 mortality has also been widely documented. Children and younger individuals are generally asymptomatic or present with mild symptoms, whereas individuals aged over 80 years and those with chronic underlying diseases are particularly vulnerable to severe outcomes (10). Notably, COVID-19 mortality increases sharply after the age of 50, with reported mortality rates of 1.3% in individuals aged 50–59 years, 3.6% in those aged 60–69 years, 8.0% in those aged 70–79 years, and 14.8% in individuals aged ≥80 years (11).

COVID-19 primarily affects the respiratory system and presents with a wide spectrum of clinical manifestations, including fever, dry cough, and dyspnea, often accompanied by fatigue, pneumonia, dizziness, and gastrointestinal symptoms (12). Patients with severe COVID-19 may develop acute respiratory distress syndrome (ARDS), acute respiratory failure, cytokine storm syndrome characterized by excessive release of cytokines and chemokines, septic shock, and multi-organ dysfunction syndrome (13–17). These pathological changes are major contributors to the high mortality rate among patients with severe COVID-19. Beyond pneumonia, COVID-19 can induce complications involving the cardiovascular, hepatic, renal, and nervous systems, and is therefore considered a multisystem inflammatory syndrome (12, 18). Many patients develop varying degrees of multi-organ dysfunction, including acute kidney injury, myocardial injury, liver dysfunction, and neurological disorders. Furthermore, individuals with pre-existing renal, cardiac, or hepatic diseases are more likely to develop severe organ-related manifestations and complications following SARS-CoV-2 infection (14, 19).

Previous studies have established that male sex and advanced age independently increase the risk of severe COVID-19 outcomes, yet the host response mechanisms driving these disparities remain incompletely characterized. Specifically, it is unclear whether sex- and age-associated differences in multi-organ dysfunction and immune dysregulation follow distinct biomarker profiles that could inform targeted management strategies. To address this gap, we conducted a retrospective study of hospitalized COVID-19 patients (December 2022–March 2023), collecting comprehensive clinical and laboratory data. We systematically examined biomarkers of immune response, inflammation, coagulation, and multi-organ dysfunction across sex and age strata to identify differential patterns that may explain the observed demographic differences in COVID-19 severity and clinical outcomes.

## 2 Materials and methods

### 2.1 Study design and patients

This retrospective cohort study included COVID-19 patients who were hospitalized and treated at the Ninth Hospital of Xi’an between December 2022 and March 2023. The diagnosis and clinical classification of COVID-19 were performed in strict accordance with the Diagnosis and Treatment Protocol for COVID-19 (Trial Version 10) issued by the National Health Commission of China (20). Patients presenting with typical COVID-19 symptoms, such as fever and cough, were confirmed by real-time reverse transcription polymerase chain reaction (RT-PCR) testing, which enabled differentiation from other respiratory infections and non-infectious diseases. Demographic characteristics (including sex and age), clinical information, and laboratory results were collected from admission until discharge or death. Laboratory data comprised hematological parameters, inflammatory markers, biomarkers of organ injury, and microbial culture results, which were measured through routine clinical testing conducted by the hospital’s clinical laboratory during hospitalization. Patients meeting any of the following criteria were excluded: (1) those with chronic inflammatory conditions, autoimmune diseases, immunodeficiency, malignancies, organ transplantation, pregnancy, hematological disorders, or coagulation dysfunction; (2) patients admitted primarily because of underlying diseases such as diabetes mellitus, cardiovascular disease, renal disease, or hepatic disease; and (3) patients with incomplete clinical or laboratory data.

**Figure 1:**
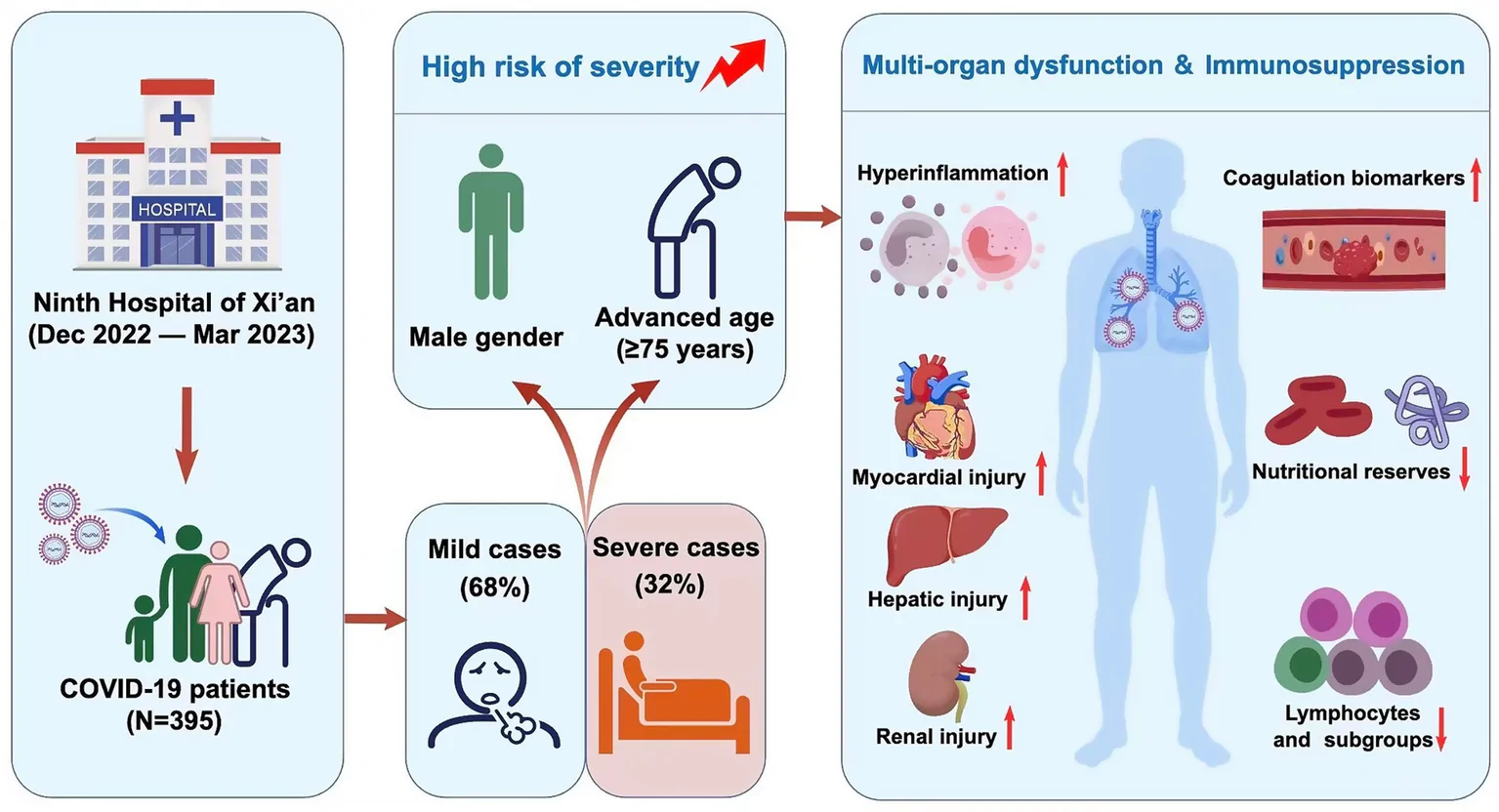
Sex and age are associated with disease severity in patients with COVID-19. Male individuals and older adults are more likely to develop exaggerated inflammatory responses, coagulation dysfunction, immunosuppression, multi-organ injury, and impaired protein synthesis, all of which contribute to unfavorable clinical outcomes. This figure was made using BioGDP (https://biogdp.com).

Based on clinical symptoms, imaging findings, and pulmonary function status, patients were categorized as mild, moderate, severe, or critical. For analytical purposes, mild and moderate cases were merged into the mild group, while severe and critical cases were combined into the severe group. Patients were further stratified by sex into female and male groups, and by age into <75-year-old and ≥75-year-old groups. The following data were systematically collected: demographic variables (sex, age, disease duration, and history of underlying diseases); laboratory parameters, including peripheral blood cell counts, immune cell and immune cell subset counts, inflammatory markers, coagulation function indices, and biomarkers of cardiac, renal, and hepatic injury (Figure 1). To ensure data quality, a dual-team independent data extraction model was implemented, in which two research teams independently collected and cross-validated the data. This study was approved by the Institutional Review Board of the Ninth Hospital of Xi’an (Approval No.: 202311) and was conducted in accordance with the principles of the Declaration of Helsinki. Given the retrospective nature of the study, the requirement for informed consent was waived by the Ethics Committee.

### 2.2 Clinical classifications

According to the guidelines for the diagnosis and management of COVID-19 (10th edition, in Chinese) released by the National Health Commission of China (20), the clinical classifications of COVID-19 are as follows. Mild cases: Clinical symptoms are mild, and there are no signs of pneumonia on imaging. Moderate cases: Patients exhibit symptoms such as fever and respiratory tract symptoms, along with pneumonia manifestation visible in imaging. Severe cases: Meet any of the following criteria — respiratory distress with a respiratory rate ≥ 30 breaths/min; SpO_2_ ≤ 93% at rest; and PaO_2_/FIO_2_ ≤ 300. Patients with over 50% lesion progression within 24 to 48 h in pulmonary imaging should be treated as severe cases. Critical cases: Those meeting one of the following conditions respiratory failure requiring mechanical ventilation, shock, or complications from other organ failures necessitating monitoring and treatment in the ICU.

### 2.3 Real-time RT-PCR for the quantification of SARS-CoV-2

Pharyngeal swab specimens were transported immediately for testing after collection or stored at 4 °C and processed within 8 h. Viral RNA was extracted in accordance with the manufacturer’s instructions. Briefly, 20 μL of extracted RNA was added to a 20 μL reaction mixture containing a commercial real-time RT-PCR master mix and specific primers (Daan Gene Co., Ltd., Guangzhou, China). The detection limit of the ORF1ab and N gene real-time RT-PCR assay was approximately 200 copies/mL. Samples with cycle threshold (Ct) values ≤40.0 were classified as positive according to the routine diagnostic criteria of the hospital clinical laboratory and the manufacturer’s instructions in effect during the study period. All RT-PCR analyses were performed using the ABI 7500 Real-Time PCR System (Applied Biosystems, Foster City, CA, United States).

### 2.4 Detection of immune response and multi-organ injury biomarkers

Blood samples were collected within 48 h after the clinical diagnosis of COVID-19, rather than at a fixed time after symptom onset; the interval from symptom onset to sampling was not recorded directly. Accordingly, the duration from symptom onset to discharge, which differed between severity groups, was included as a covariate in multivariable analyses. Peripheral blood was drawn into EDTA anticoagulant tubes for hematological analysis. Blood cells were stained using a hematology staining reagent and analyzed with the CAL8000 automated hematology analyzer (Mindray Co., Ltd., Shenzhen, China). Serum samples were collected in separation gel tubes. Hypersensitive C-reactive protein (hs-CRP) and serum amyloid A (SAA) were measured using the BN II analyzer (Siemens, Munich, Germany). Interleukin-6 (IL-6), procalcitonin (PCT), N-terminal pro–brain natriuretic peptide (NT-proBNP), and hypersensitive cardiac troponin T (hs-TnT) were determined using the Cobas e411 electrochemiluminescence immunoassay system (Roche, Basel, Switzerland). Serum proteins and biomarkers of cardiac, hepatic, and renal injury, including total protein (TP), albumin (ALB), aspartate aminotransferase (AST), mitochondrial aspartate aminotransferase (m-AST), creatine kinase (CK), creatine kinase-MB (CK-MB), lactate dehydrogenase (LDH), α-hydroxybutyrate dehydrogenase (HBDH), ischemia-modified albumin (IMA), total bilirubin (TBIL), direct bilirubin (DBIL), alanine aminotransferase (ALT), urea (UREA), creatinine (CREA), and cystatin C (CYSC), were measured using reagents from Meikang Co., Ltd. (Ningbo, China) on a TBA-FX8 biochemical analyzer (Canon Medical Systems, Tochigi, Japan). Additional blood samples collected in sodium citrate anticoagulant tubes were used to assess coagulation parameters, including prothrombin time (PT), activated partial thromboplastin time (APTT), thrombin time (TT), fibrinogen (FIB), D-dimer, and fibrinogen degradation products (FDP), using the STA-R Max coagulation analyzer (Diagnostica Stago, Asnières-sur-Seine, France).

### 2.5 Flow cytometric analysis of peripheral blood TBNK lymphocyte subsets

For lymphocyte subset analysis, peripheral blood samples were collected in EDTA-anticoagulant tubes and analyzed by flow cytometry. T-cell subsets were assessed using a four-color antibody panel consisting of CD45-FITC, CD4-PE, CD8-ECD, and CD3-PC5.5. Lymphocytes were first identified as CD45^bright^/SSC^low^ cells (R1) on a CD45 versus side-scatter (SSC) plot. Total T cells were subsequently identified as CD3^+^ cells within the R1 gate (R2). CD4^+^ and CD8^+^ T-cell subsets were determined by two-parameter analysis of CD3 versus CD4 and CD3 versus CD8, respectively, and defined as CD3^+^CD4^+^ and CD3^+^CD8^+^ cells. B cells and natural killer (NK) cells were analyzed using a five-color antibody panel consisting of CD45-FITC, CD56-PE, CD19-ECD, CD3-PC5.5, and CD16-PE. Following identification of the lymphocyte population using the CD45 versus SSC plot (R1), CD3^−^ cells were gated to exclude T cells. Within the CD3^−^ population, CD19^+^ cells were identified as B cells, whereas NK cells were defined as CD56^+^ and/or CD16^+^ cells. For absolute cell counting, a known concentration of Flow-Count fluorescent beads (Beckman Coulter, Indianapolis, IN, United States) was added to each tube before antibody staining. Flow cytometric data were analyzed using Kaluza software version 2.1 (Beckman Coulter, Indianapolis, IN, United States). A viability dye was not included in the predefined antibody panels used in this retrospective clinical testing workflow. Lymphocyte populations were identified using the CD45/SSC gating strategy, with lymphocytes defined as CD45^bright^/SSC^low^ events. We acknowledge that this approach cannot completely distinguish viable cells from apoptotic or dead cells. To minimize the potential impact of compromised sample quality, all specimens included in the analysis underwent standardized pre-analytical quality control, including processing within 24 h of sample collection and exclusion of samples with obvious hemolysis or coagulation.

### 2.6 Microbial culture and pathogen identification

Microbiological specimens included blood, sputum, and urine samples. Blood samples were inoculated into aerobic and anaerobic culture bottles and incubated in the BacT/ALERT 3D automated blood culture system (bioMérieux, Marcy-l’Étoile, France). Positive broth cultures were subsequently subcultured onto appropriate solid media for pathogen isolation. Deep sputum and midstream urine specimens were cultured on blood agar, MacConkey agar, and chocolate agar plates to isolate potential pathogens. Microbial identification was performed using the VITEK 2 Compact automated microbial identification system (bioMérieux, Marcy-l’Étoile, France) and matrix-assisted laser desorption/ionization time-of-flight mass spectrometry (MALDI-TOF MS; Bruker Daltonics, Bremen, Germany).

### 2.7 Statistical analysis

Statistical analyses were conducted using GraphPad Prism (version 9.5.0) and SPSS (version 27). Continuous variables were first assessed for normality with the Shapiro–Wilk test. Student’s *t*-test was used when both groups satisfied normality (*p* ≥ 0.05); otherwise, the Mann–Whitney *U* test was used. Categorical variables were compared using the *χ*^2^ test or Fisher’s exact test, as appropriate. To control for multiple comparisons across the 45 biomarkers, the Benjamini–Hochberg (BH) procedure was applied to adjust the false discovery rate (FDR) in each set of comparisons, and adjusted *p*-values (*q* values) < 0.05 were considered significant. Spearman’s rank correlation was used to describe associations of sex and age with biomarker levels; the resulting correlations were also corrected for multiple testing using the same FDR approach (*q* < 0.05). Multivariable logistic regression was used to estimate adjusted odds ratios (ORs) and 95% confidence intervals (CIs) for severe COVID-19. A two-sided *p* < 0.05 was considered statistically significant in regression models.

## 3 Results

### 3.1 Basic information of hospitalized COVID-19 patients

**Figure 2:**
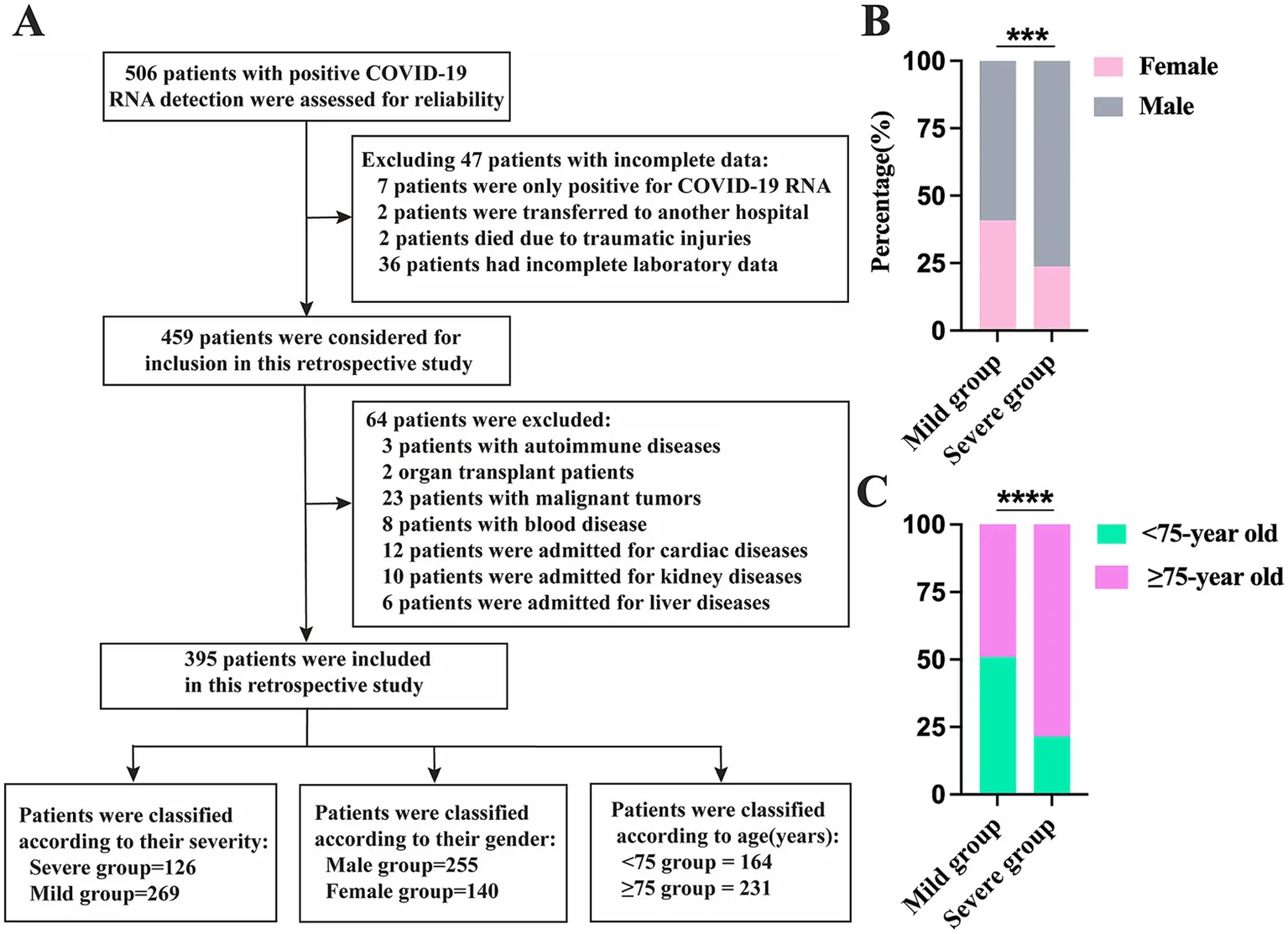
Demographic and clinical characteristics of 395 COVID-19 patients. **(A)** Flowchart of patient inclusion and exclusion criteria. **(B)** Comparison of sex distribution percentages between the severe and mild groups. **(C)** Comparison of age distribution percentages between the severe and mild groups. Categorical variables were compared with the χ^2^ test or Fisher’s exact test; continuous variables were compared with Student’s *t* or Mann–Whitney *U* test after Shapiro–Wilk normality assessment. *p* < 0.05 was considered significant. ***p < 0.001, ****p < 0.0001.

To investigate the relationship between age, sex, and the severity and hospitalization rate of COVID-19, this retrospective cohort study included 395 hospitalized COVID-19 patients, with the inclusion and exclusion criteria shown in Figure 2A. All patients underwent chest CT scans and SARS-CoV-2 RNA tests upon admission, SARS-CoV-2 infection was confirmed by RT-PCR, and chest CT findings were incorporated into the final clinical classification according to the national protocol. Among the 395 patients, 269 (68%) had mild cases, while 126 (32%) had severe cases; 255 (65%) were male and 140 (35%) were female; 231 (58%) were aged ≥75 years, and 164 (42%) were aged <75 years. The hospitalization rate was significantly higher among males and patients aged ≥75 years. Compared to the mild group, the severe group was significantly older, particularly with the ≥75-year-old patients constituting the majority of the severe group (79%). There was no significant age difference between male and female patients, but the proportion of male patients (76%) was notably higher in the severe group than that of females, as shown in Figures 2B,C and Table 1. The mean duration from symptom onset to discharge was significantly longer in severe cases (23.83 days) compared to mild cases (11.58 days), in male patients (16.51 days) compared to female patients (13.63 days), and in patients aged ≥75 years (17.83 days) compared to those aged <75 years (12.2 days), as shown in Table 1. To determine whether these demographic and clinical differences were independently associated with severity, multivariable logistic regression was performed (Table 2). After adjustment (Model 1), co-infection (OR 15.665, 95% CI 7.739–31.709, *p* < 0.001) and kidney disease (OR 4.485, 95% CI 2.042–9.851, *p* < 0.001) were independently associated with severe COVID-19, whereas cardiovascular disease, hypertension, type 2 diabetes, and pulmonary disease were not significant. When days from symptom onset to discharge was added to the model (Model 2), longer duration was independently associated with severity (OR 1.420 per day, 95% CI 1.311–1.537, *p* < 0.001). The adjusted estimates for male sex and age ≥75 years are detailed in Sections 3.3 and 3.4.

**Table 1:** Demographics and clinical characteristics of patients with COVID-19.

Clinical characteristics	Mild cases (*n* = 269)	Severe cases (*n* = 126)	*P*	Male cases (*n* = 255)	Female cases (*n* = 140)	*P*	<75-year old cases (*n* = 164)	≥75-year old cases (*n* = 231)	*P*
Age, mean, years (± SD)	72.94 (13.33)	80.01 (9.90)	<0.0001	74.86 (12.07)	75.80 (13.96)	0.1048	63.29 (10.51)	83.65 (5.27)	<0.0001
Days from symptom onset to discharge (± SD)	11.58 (4.75)	23.83 (6.86)	<0.0001	16.51 (8.20)	13.63 (7.09)	0.0005	12.20 (6.94)	17.83 (7.78)	<0.0001
Underlying diseases									
Cardiovascular disease, *n* (%)	132 (49.07)	81 (64.29)	0.005	144 (56.47)	69 (49.29)	0.171	64 (39.02)	149 (64.50)	<0.0001
Hypertension, *n* (%)	156 (57.99)	81 (64.29)	0.234	162 (63.53)	75 (53.57)	0.053	79 (48.17)	158 (68.40)	<0.0001
Type 2 diabetes, *n* (%)	87 (32.34)	35 (27.78)	0.36	82 (32.16)	40 (28.57)	0.461	54 (32.93)	68 (29.44)	0.459
Kidney diseases, *n* (%)	16 (5.95)	29 (23.02)	<0.0001	38 (14.90)	7 (5.00)	0.003	15 (9.15)	30 (12.99)	0.236
Pulmonary diseases, *n* (%)	44 (16.36)	26 (20.63)	0.299	58 (22.75)	12 (8.57)	0.0004	25 (15.24)	45 (19.48)	0.277
Cerebrovascular diseases, *n* (%)	28 (10.41)	17 (13.49)	0.369	27 (10.59)	18 (12.86)	0.497	11 (6.71)	34 (14.72)	0.014
Liver diseases, *n* (%)	10 (3.72)	17 (13.49)	0.0003	24 (9.41)	3 (2.14)	0.006	11 (6.71)	16 (6.93)	0.932
Alzheimer’s disease, *n* (%)	0 (0)	7 (5.56)	<0.0001	2 (0.78)	5 (3.57)	0.045	1 (0.61)	6 (2.60)	0.14
Parkinson’s disease, *n* (%)	4 (1.49)	6 (4.76)	0.053	8 (3.14)	2 (1.43)	0.301	1 (0.61)	9 (3.90)	0.04
Co-infection, *n* (%)	15 (5.58)	57 (45.24)	<0.0001	57 (22.35)	15 (10.71)	0.004	25 (15.24)	47 (20.35)	0.196

Categorical variables are presented as *n* (%) and were compared with the *χ*^2^ test or Fisher’s exact test; continuous variables are presented as mean ± standard deviation (SD) and were compared with Student’s *t* or Mann–Whitney *U* test after Shapiro–Wilk normality assessment. *p* < 0.05 was considered significant.

**Table 2:** Multivariable logistic regression analysis of factors associated with severe COVID-19.

Variable	Model 1 OR (95% CI)	*P*	Model 2 OR (95% CI)	*P*
Male sex (vs. female)	1.925 (1.063–3.484)	0.031	1.463 (0.640–3.346)	0.367
Age ≥75 years (vs. <75 years)	5.164 (2.733–9.756)	<0.001	2.312 (0.940–5.687)	0.068
Co-infection (yes vs. no)	15.665 (7.739–31.709)	<0.001	9.600 (3.564–25.858)	<0.001
Cardiovascular disease	1.096 (0.633–1.901)	0.743	1.360 (0.633–2.920)	0.430
Hypertension	1.066 (0.599–1.896)	0.827	0.630 (0.284–1.397)	0.256
Type 2 diabetes	0.681 (0.376–1.231)	0.203	0.557 (0.248–1.253)	0.157
Kidney disease	4.485 (2.042–9.851)	<0.001	5.843 (1.872–18.237)	0.002
Pulmonary disease	1.152 (0.588–2.258)	0.679	0.603 (0.231–1.569)	0.300
Days from symptom onset to discharge (per day)			1.420 (1.311–1.537)	<0.001

OR, odds ratio; CI, confidence interval. Model 1 included sex, age, co-infection, cardiovascular disease, hypertension, type 2 diabetes, kidney disease, and pulmonary disease. Model 2 additionally included days from symptom onset to discharge. Sensitivity analyses excluding co-infected patients (*n* = 323; events = 69) yielded OR 2.136 (95% CI 1.111–4.107, *p* = 0.023) for male sex and OR 3.230 (95% CI 1.656–6.301, *p* < 0.001) for age ≥75 years. In patients with ≤20 days from symptom onset to discharge (*n* = 304; events = 42), the corresponding ORs were 2.675 (95% CI 1.105–6.473, *p* = 0.029) and 4.468 (95% CI 1.759–11.351, *p* = 0.002).

Most patients presented with underlying comorbidities, among which hypertension was the most prevalent. In the context of the COVID-19 pandemic, the proportions of renal disease, cardiovascular disease, hepatic disease, and Alzheimer’s disease were significantly higher in severe cases than in mild cases. Sex-stratified analysis demonstrated that male patients had significantly higher rates of renal disease, hepatic disease, and pulmonary disease compared with female patients, whereas Alzheimer’s disease was more prevalent among female patients. Age-stratified analysis further revealed that patients aged ≥75 years had significantly higher proportions of hypertension, cardiovascular disease, cerebrovascular disease, and Parkinson’s disease compared with those aged <75 years. In addition, the incidence of co-infections was higher in severe cases than in mild cases, and was also higher in male patients than in female patients. Detailed data are presented in Table 1.

### 3.2 Biomarker profile associated with COVID-19 severity

To identify biomarkers associated with COVID-19 severity, we systematically analyzed 45 laboratory parameters in patients with mild and severe disease. After FDR correction, 26 biomarkers were significantly elevated, whereas 11 biomarkers were significantly reduced in severe cases compared with mild cases (Table 3). Regarding immune response characteristics, severe cases exhibited significantly higher WBC and neutrophil counts, NLR, hs-CRP, PCT, IL-6, and SAA levels, accompanied by significantly lower lymphocyte counts. In addition, the absolute counts of CD3^+^ T cells, CD4^+^ T cells, CD8^+^ T cells, B cells, and NK cells were significantly reduced in severe cases. However, no significant differences were observed in the percentages of CD3^+^ T cells, CD4^+^ T cells, CD8^+^ T cells, B cells, or NK cells between the mild and severe groups. In terms of multi-organ dysfunction, coagulation parameters PT, APTT, TT, FIB, D-dimer, and FDP were significantly elevated. Myocardial injury markers hs-TNT, NT-proBNP, CK-MB, LDH, HBDH, and IMA were significantly higher, whereas CK was not significant after FDR correction. Hepatic injury markers AST, m-AST, DBIL, and ALT were significantly higher, and renal injury markers UREA, CREA, and CYSC were also increased. RBC count, HGB, HCT, TP, and ALB were significantly lower in severe cases. Collectively, these findings indicate that severe COVID-19 is associated with pronounced immunosuppression and widespread multi-organ dysfunction, which likely contribute to disease progression and increased severity.

**Table 3:** Comparison of laboratory parameters between mild and severe patients with COVID-19.

Biomarker name	Reference range	Mild cases (n)	Severe cases (n)	Mild cases Mean (SD)	Severe cases Mean (SD)	*P*	FDR *q*	FDR-significant
Inflammatory markers								
WBC, ×10^9^/L	4.1–11.0	269	126	5.542(1.832)	9.162(5.325)	<0.0001	<0.0001	Y
Neutrophil, ×10^9^/L	1.8–8.3	269	126	3.932(1.724)	8.096(5.218)	<0.0001	<0.0001	Y
Monocyte, ×10^9^/L	0.14–0.74	269	125	0.4056(0.1704)	0.4162(0.2499)	0.2457	0.2691	N
NLR		269	126	4.294 (3.358)	20.12 (42.25)	<0.0001	<0.0001	Y
hs-CRP, mg/L	0–3.0	261	125	17.98(23.55)	107.9(65.02)	<0.0001	<0.0001	Y
PCT, ng/mL	0–0.046	214	107	0.08730(0.1587)	0.6112(0.9158)	<0.0001	<0.0001	Y
IL-6, pg./mL	0–7	149	85	17.19(20.24)	107.4(100.2)	<0.0001	<0.0001	Y
SAA, mg/L	0–6.4	101	58	109.4(150.2)	481.8(308.4)	<0.0001	<0.0001	Y
Coagulation markers								
PT, s	11–14	261	124	12.97(1.862)	15.08(2.999)	<0.0001	<0.0001	Y
APTT, s	28.0–43.5	259	124	38.17(5.729)	44.76(10.61)	<0.0001	<0.0001	Y
TT, s	14–21	261	123	17.91(2.364)	18.73(3.010)	0.0169	0.0174	Y
FIB, g/L	2.00–4.00	259	124	4.114(1.224)	4.864(1.588)	<0.0001	<0.0001	Y
D-dimer, μg/mL	0–0.5	246	121	0.8918(0.7928)	4.069(4.995)	<0.0001	<0.0001	Y
FDP, μg/mL	0–5.0	246	120	2.761(2.741)	15.50(20.39)	<0.0001	<0.0001	Y
Myocardial injury markers								
CK, U/L	50–310	189	103	97.31(92.66)	165.3(215.3)	0.2811	0.3008	N
CK-MB, U/L	0–24	185	101	13.24(5.628)	16.81(9.266)	0.0039	0.0042	Y
LDH, U/L	120–250	189	102	220.8(63.21)	315.7(165.0)	<0.0001	<0.0001	Y
HBDH, U/L	72–182	189	103	174.2(54.02)	246.4(128.5)	<0.0001	<0.0001	Y
IMA, U/mL	0–85	189	105	83.27(4.615)	89.88(4.134)	<0.0001	<0.0001	Y
hs-TNT, ng/mL	0–0.0140	150	89	0.01957(0.02137)	0.08948(0.1070)	<0.0001	<0.0001	Y
NT-proBNP, pg./mL	0–900	140	92	855.8(1563)	6,165(9353)	<0.0001	<0.0001	Y
Liver injury markers								
TBIL, μmol/L	0–26	264	122	11.97(5.512)	12.53(7.461)	0.8814	0.9019	N
DBIL, μmol/L	0–8	263	120	4.489(2.202)	5.581(3.062)	0.0012	0.0014	Y
AST, U/L	15–40	198	102	25.57(12.97)	55.68(51.69)	<0.0001	<0.0001	Y
m-AST, U/L	0–15.0	193	101	8.576(4.671)	17.81(15.07)	<0.0001	<0.0001	Y
ALT, U/L	9–50	264	118	24.76(19.44)	31.47(23.58)	0.0057	0.0060	Y
Renal injury markers								
UREA, mmol/L	3.1–8.0	263	123	5.847(2.894)	13.41(9.069)	<0.0001	<0.0001	Y
CREA, mmol/L	57–97	264	122	72.75(27.62)	130.0(100.8)	<0.0001	<0.0001	Y
CYSC, mg/l	0.51–1.09	246	122	1.199(0.4106)	2.190(1.455)	<0.0001	<0.0001	Y
Nutritional and anemia-related markers								
RBC, ×10^12^/L	4.3–5.8	269	126	3.994(0.5870)	3.685(0.6886)	<0.0001	<0.0001	Y
Hemoglobin, g/L	130–175	269	126	124.5(18.92)	114.3(21.99)	<0.0001	<0.0001	Y
Hematocrit, L/L	0.40–0.50	269	126	0.3677(0.05088)	0.3368(0.06062)	<0.0001	<0.0001	Y
TP, g/L	65.0–85.0	266	123	60.75(5.803)	54.34(5.596)	<0.0001	<0.0001	Y
ALB, g/L	40.0–55.0	266	124	35.38(4.715)	28.23(4.346)	<0.0001	<0.0001	Y
Cellular immunological markers								
Lymphocyte, ×10^9^/L	1.2–3.8	269	126	1.120(0.4901)	0.6051(0.3345)	<0.0001	<0.0001	Y
CD3^+^T, ×10^6^/L	723–2,271	37	37	818.6 (278.2)	321.1(214.2)	<0.0001	<0.0001	Y
CD3^+^CD4^+^T, ×10^6^/L	396–1,309	36	37	460.6(181.6)	179.8(131.4)	<0.0001	<0.0001	Y
CD3^+^CD8^+^T, ×10^6^/L	224–1,014	36	37	326.6(172.5)	127.50(104.4)	<0.0001	<0.0001	Y
B, ×10^6^/L	118–645	36	36	110.5(68.73)	55.50(57.53)	0.0004	0.0006	Y
NK, ×10^6^/L	61–607	35	36	181.3(114.9)	103.9(76.48)	0.0013	0.0017	Y
CD3^+^T %	61.1–77	37	37	72.99 (10.33)	65.88 (15.58)	0.0637	0.0716	N
CD3^+^CD4^+^T %	25.8–41.6	36	37	42.24 (9.221)	37.42 (14.15)	0.0535	0.0631	N
CD3^+^CD8^+^T %	18.1–29.6	36	37	28.43 (10.22)	25.96 (11.64)	0.3394	0.3548	N
B %	9.02–14.1	36	36	9.453 (5.513)	10.71 (8.294)	0.4521	0.4622	N
NK %	10.04–19.78	35	36	15.77 (9.28)	20.85 (13.07)	0.0638	0.0716	N

Results are expressed as mean ± standard deviation (SD); Normality was assessed with the Shapiro–Wilk test; Student’s *t*-test was used when both groups satisfied normality, and the Mann–Whitney *U* test otherwise. *P* and FDR *q* values are shown; *q* < 0.05 was considered significant. Benjamini–Hochberg FDR correction was applied across all 45 markers within this comparison. WBC, White blood cell count; NLR: Neutrophil/Lymphocyte; hs-CRP, Hypersensitive C-reactive protein; SAA, serum amyloid A; IL-6, Interleukin-6; PCT, procalcitonin; PT, prothrombin time; APTT, activated partial thromboplastin time; TT, thrombin time; FIB, fibrinogen; FDP, fibrinogen degradation products; CK, creatine kinase; CK-MB, creatine kinase-MB; LDH, lactate dehydrogenase; HBDH, α-hydroxybutyrate dehydrogenase; IMA, ischemia-modified albumin; NT-proBNP, N-terminal pro–brain natriuretic peptide; hs-TnT, hypersensitive cardiac troponin T; DBIL, direct bilirubin; AST, aspartate aminotransferase; m-AST, mitochondrial aspartate aminotransferase; ALT, alanine aminotransferase; UREA, urea; CREA, creatinine; CYSC, cystatin C; RBC, red blood cell count; HGB, hemoglobin; HCT, hematocrit; TP, total protein; ALB, albumin.

### 3.3 Sex differences in host response biomarkers and COVID-19 severity

To elucidate sex differences in the clinical severity of COVID-19, we compared host response biomarker profiles between male and female patients. After FDR correction, 24 biomarkers differed significantly, with 21 elevated and 3 biomarkers were significantly reduced in male patients compared with female patients (Table 4). Male patients had significantly higher WBC and neutrophil counts, NLR, hs-CRP, PCT, and IL-6 levels, and lower lymphocyte counts. Absolute counts and percentages of CD3^+^ T, CD4^+^ T, CD8^+^ T, B, and NK cells did not differ significantly between sexes. Although not statistically significant, T, B, or NK cell counts showed decreasing trends in males. Coagulation markers PT, APTT, and FIB were significantly higher in males, whereas TT, D-dimer, and FDP did not differ. Myocardial injury markers CK, IMA, and hs-TNT were significantly higher in males, whereas CK-MB was not significant after FDR correction. Hepatic injury markers DBIL, AST, m-AST, and ALT were higher in males; TBIL was not significant. Renal injury markers UREA, CREA, and CYSC were higher. HGB and HCT were higher in males, while TP and ALB were lower; RBC did not differ significantly after FDR correction. In multivariable logistic regression, male sex remained associated with severe COVID-19 (OR 1.925, 95% CI 1.063–3.484, *p* = 0.031) after adjustment, and the association persisted in sensitivity analyses excluding co-infected patients (OR 2.136, 95% CI 1.111–4.107, *p* = 0.023; Table 2).

**Table 4:** Comparison of laboratory parameters between male and female patients with COVID-19.

Biomarker name	Reference range	Male cases (n)	Female cases (n)	Male cases Mean (SD)	Female cases Mean (SD)	*P*	FDR *q*	FDR-significant
Inflammatory markers								
WBC, ×10^9^/L	4.1–11.0	255	140	7.061(4.111)	6.033(2.914)	0.0198	0.0434	Y
Neutrophil, ×10^9^/L	1.8–8.3	255	140	5.663(4.154)	4.525(2.924)	0.0060	0.0172	Y
NLR		255	140	10.91 (30.26)	6.479 (9.763)	0.0001	0.0008	Y
Monocyte, ×10^9^/L	0.14–0.74	254	140	0.4165(0.2032)	0.3952(0.1906)	0.3089	0.4306	N
hs-CRP, mg/L	0–3.0	248	138	55.48(64.85)	32.04(43.93)	0.0007	0.0030	Y
PCT, ng/mL	0–0.046	211	110	0.3064(0.6373)	0.1768(0.5010)	0.0001	0.0008	Y
IL-6, pg./mL	0–7	157	77	57.25(80.75)	35.13(63.01)	0.0110	0.0266	Y
SAA, mg/L	0–6.4	108	51	274.2(299.1)	183.9(242.0)	0.0614	0.1149	N
Coagulation markers								
PT, s	11–14	247	138	13.80(2.273)	13.39(2.825)	0.0022	0.0066	Y
APTT, s	28.0–43.5	246	137	41.53(8.286)	38.10(7.725)	<0.0001	0.0008	Y
TT, s	14–21	247	137	18.17(2.698)	18.16(2.460)	0.9581	0.9581	N
FIB, g/L	2.00–4.00	246	137	4.500(1.474)	4.100(1.205)	0.0080	0.0206	Y
D-dimer, μg/mL	0–0.5	237	130	2.018(3.408)	1.796(3.077)	0.5364	0.6389	N
FDP, μg/mL	0–5.0	236	130	7.228(13.30)	6.413(13.29)	0.5753	0.6616	N
Myocardial injury markers								
CK, U/L	50–310	184	108	133.9(153.5)	99.78(145.4)	0.0037	0.0098	Y
CK-MB, U/L	0–24	182	104	15.10(7.409)	13.45(7.098)	0.0665	0.1177	N
LDH, U/L	120–250	184	107	263.0(131.6)	238.6(91.57)	0.0924	0.1524	N
HBDH, U/L	72–182	184	108	203.5(98.18)	193.3(86.91)	0.3727	0.5043	N
IMA, U/mL	0–85	186	108	86.51(5.139)	84.10(5.679)	0.0002	0.0013	Y
hs-TNT, ng/mL	0–0.0140	152	87	0.04703(0.06544)	0.04311(0.09033)	0.0076	0.0170	Y
NT-proBNP, pg./mL	0–900	153	79	3,173(6748)	2,552(6122)	0.4941	0.6242	N
Liver injury markers								
TBIL, μmol/L	0–26	245	138	12.76(6.800)	11.04(4.734)	0.0337	0.0704	N
DBIL, μmol/L	0–8	188	112	5.243(2.725)	4.100(2.015)	<0.0001	<0.0001	Y
AST, U/L	15–40	185	109	40.48(40.99)	27.95(18.68)	0.0005	0.0024	Y
m-AST, U/L	0–15.0	244	138	12.88(11.92)	9.828(7.299)	0.0133	0.0307	Y
ALT, U/L	9–50	245	138	30.63(23.58)	20.11(13.00)	<0.0001	<0.0001	Y
Renal injury markers								
UREA, mmol/L	3.1–8.0	249	137	9.405(7.419)	6.175(4.251)	<0.0001	<0.0001	Y
CREA, mmol/L	57–97	248	138	102.1(70.47)	70.52(53.21)	<0.0001	<0.0001	Y
CYSC, mg/l	0.51–1.09	238	130	1.659(1.148)	1.287(0.6437)	0.0001	0.0008	Y
Nutritional and anemia-related markers								
RBC, ×10^12^/L	4.3–5.8	255	140	3.944(0.6692)	3.807(0.5648)	0.0397	0.0795	N
Hemoglobin, g/L	130–175	255	140	123.5(21.49)	117.1(17.84)	0.0028	0.0099	Y
Hematocrit, L/L	0.40–0.50	255	140	0.3638(0.05840)	0.3470(0.04969)	0.0042	0.0129	Y
TP, g/L	65.0–85.0	251	138	58.07(6.350)	59.91(6.516)	0.0073	0.0196	Y
ALB, g/L	40.0–55.0	251	139	32.45(5.786)	34.29(5.290)	0.0021	0.0079	Y
Cellular immunological markers								
Lymphocyte, ×10^9^/L	1.2–3.8	255	140	0.9015(0.4886)	1.054(0.5252)	0.0042	0.0129	Y
CD3^+^T, ×10^6^/L	723–2,271	45	29	501.0(318.8)	637.3(335.7)	0.1865	0.2768	N
CD3^+^CD4^+^T, ×10^6^/L	396–1,309	44	29	295.5(208.8)	368.5(206.9)	0.1467	0.2249	N
CD3^+^CD8^+^T, ×10^6^/L	224–1,014	44	29	215.6(185.2)	241.1(154.8)	0.5416	0.6389	N
B, ×10^6^/L	118–645	43	29	75.30(65.64)	94.45(72.79)	0.2493	0.3584	N
NK, ×10^6^/L	61–607	42	29	134.9(106.1)	152.5(102.3)	0.4872	0.6242	N
CD3^+^T %	61.1–77	45	29	68.58 (13.67)	70.77 (13.64)	0.5021	0.6242	N
CD3^+^CD4^+^T %	25.8–41.6	44	29	39.45 (12.95)	40.32 (11.01)	0.7672	0.8006	N
CD3^+^CD8^+^T %	18.1–29.6	44	29	26.86 (11.45)	27.66 (10.34)	0.7611	0.8006	N
B %	9.02–14.1	43	29	10.35 (8.072)	9.686 (5.195)	0.6983	0.7667	N
NK %	10.04–19.78	42	29	18.67 (11.52)	17.89 (11.83)	0.7832	0.8006	N

Results are expressed as mean ± standard deviation (SD); Normality was assessed with the Shapiro–Wilk test; Student’s *t*-test was used when both groups satisfied normality, and the Mann–Whitney *U* test otherwise. *P* and FDR *q* values are shown; *q* < 0.05 was considered significant. Benjamini–Hochberg FDR correction was applied across all 45 markers within this comparison. WBC, White blood cell count; NLR, Neutrophil/Lymphocyte; hs-CRP, Hypersensitive C-reactive protein; SAA, serum amyloid A; IL-6, Interleukin-6; PCT, procalcitonin; PT, prothrombin time; APTT, activated partial thromboplastin time; TT, thrombin time; FIB, fibrinogen; FDP, fibrinogen degradation products; CK, creatine kinase; CK-MB, creatine kinase-MB; LDH, lactate dehydrogenase; HBDH, α-hydroxybutyrate dehydrogenase; IMA, ischemia-modified albumin; NT-proBNP, N-terminal pro–brain natriuretic peptide; hs-TnT, hypersensitive cardiac troponin T; DBIL, direct bilirubin; AST, aspartate aminotransferase; m-AST, mitochondrial aspartate aminotransferase; ALT, alanine aminotransferase; UREA, urea; CREA, creatinine; CYSC, cystatin C; RBC, red blood cell count; HGB, hemoglobin; HCT, hematocrit; TP, total protein; ALB, albumin.

### 3.4 Age-related disparities in host response biomarkers and COVID-19 severity

To investigate age-related disparities in COVID-19 severity, host response biomarker profiles were systematically compared across different age groups. After FDR correction, 22 biomarkers differed significantly, with 16 elevated and 6 reduced in patients aged ≥75 years (Table 5). Older patients had significantly higher NLR, hs-CRP, IL-6, PCT, and SAA levels and lower lymphocyte counts. Absolute counts and percentages of CD3^+^ T, CD4^+^ T, CD8^+^ T, B, and NK cells did not differ significantly between age groups after FDR correction. Although not statistically significant, T, B, or NK cell counts showed decreasing trends in patients aged ≥75 years. Coagulation markers PT, APTT, FIB, D-dimer, and FDP were significantly higher in older patients; TT did not differ. Myocardial injury markers IMA, hs-TNT, and NT-proBNP were significantly higher, whereas CK, CK-MB, LDH, and HBDH were not significant after FDR correction. Hepatic markers were not significantly different after FDR correction. Renal injury markers UREA, CREA, and CYSC were significantly higher. RBC count, HGB, HCT, TP, and ALB were significantly lower in older patients. In multivariable analysis, age ≥75 years was associated with severe COVID-19 (OR 5.164, 95% CI 2.733–9.756, *p* < 0.001), and the association remained significant after excluding co-infected patients (OR 3.230, 95% CI 1.656–6.301, *p* < 0.001) and in patients with onset-to-discharge ≤20 days (OR 4.468, 95% CI 1.759–11.351, *p* = 0.002; Table 2). Overall, older patients with COVID-19 demonstrated more pronounced immunosuppression and multi-organ dysfunction, which may underlie their increased clinical risk and poorer prognosis.

**Table 5:** Comparison of laboratory parameters between COVID-19 patients aged <75 years and those aged ≥75 years.

Biomarker name	Reference range	<75-year old cases (n)	≥75-year old cases (n)	<75-year old cases mean (SD)	≥75-year old cases mean (SD)	*P*	FDR *q*	FDR-significant
Inflammatory markers								
WBC, ×10^9^/L	4.1–11.0	164	231	6.449(3.388)	6.873(4.000)	0.6384	0.6989	N
Neutrophil, ×10^9^/L	1.8–8.3	164	231	4.857(3.466)	5.546(4.003)	0.0908	0.1647	N
NLR		164	231	6.204 (7.566)	11.57 (32)	<0.0001	0.0001	Y
Monocyte, ×10^9^/L	0.14–0.74	164	230	0.4012(0.1850)	0.4145(0.2084)	0.4295	0.5645	N
hs-CRP, mg/L	0–3.0	160	226	35.10(51.30)	55.60(63.04)	<0.0001	<0.0001	Y
PCT, ng/mL	0–0.046	129	192	0.2087(0.5042)	0.2959(0.6475)	0.0043	0.0092	Y
IL-6, pg./mL	0–7	94	140	40.15(70.05)	56.11(79.05)	0.0002	0.0004	Y
SAA, mg/L	0–6.4	57	102	184.0(235.9)	280.4(304.5)	0.0010	0.0024	Y
Coagulation markers								
PT, s	11–14	157	228	12.94(1.897)	14.14(2.723)	<0.0001	<0.0001	Y
APTT, s	28.0–43.5	156	227	38.04(6.275)	41.86(9.053)	<0.0001	0.0002	Y
TT, s	14–21	157	227	17.83(2.493)	18.40(2.672)	0.0931	0.1647	N
FIB, g/L	2.00–4.00	156	227	4.229(1.428)	4.445(1.369)	0.0222	0.0485	Y
D-dimer, μg/mL	0–0.5	144	223	1.393(3.000)	2.296(3.429)	0.0123	0.0284	Y
FDP, μg/mL	0–5.0	143	223	4.475(8.821)	8.536(15.31)	<0.0001	<0.0001	Y
Myocardial injury markers								
CK, U/L	50–310	118	174	93.09(76.66)	139.1(181.2)	0.5712	0.6737	N
CK-MB, U/L	0–24	118	168	13.98(6.106)	14.84(8.026)	0.9685	0.9685	N
LDH, U/L	120–250	117	174	234.3(82.12)	266.4(135.7)	0.4981	0.6193	N
HBDH, U/L	72–182	118	174	186.5(81.58)	208.0(100.6)	0.6533	0.6989	N
IMA, U/mL	0–85	118	176	83.26(5.815)	87.11(4.665)	<0.0001	<0.0001	Y
hs-TNT, ng/mL	0–0.0140	100	139	0.02105(0.04138)	0.06124(0.08710)	<0.0001	<0.0001	Y
NT-proBNP, pg./mL	0–900	87	145	2065(6320)	3,433(6617)	<0.0001	<0.0001	Y
Liver injury markers								
TBIL, μmol/L	0–26	162	224	11.93(6.423)	12.30(6.027)	0.7425	0.7590	N
DBIL, μmol/L	0–8	161	222	4.314(2.239)	5.199(2.694)	0.0750	0.1501	N
AST, U/L	15–40	123	177	31.39(26.02)	38.70(39.46)	0.3368	0.4557	N
m-AST, U/L	0–15.0	120	174	10.54(8.127)	12.54(11.81)	0.2412	0.3579	N
ALT, U/L	9–50	160	222	30.12(23.28)	24.46(18.90)	0.1197	0.1967	N
Renal injury markers								
UREA, mmol/L	3.1–8.0	161	225	7.015(6.211)	9.149(6.824)	<0.0001	<0.0001	Y
CREA, mmol/L	57–97	160	226	81.95(63.69)	97.07(67.86)	0.0016	0.0039	Y
CYSC, mg/l	0.51–1.09	146	222	1.338(1.147)	1.652(0.8974)	<0.0001	<0.0001	Y
Nutritional and anemia-related markers								
RBC, ×10^12^/L	4.3–5.8	164	231	4.040(0.6195)	3.793(0.6303)	<0.0001	<0.0001	Y
Hemoglobin, g/L	130–175	164	231	126.8(18.88)	117.2(20.68)	<0.0001	<0.0001	Y
Hematocrit, L/L	0.40–0.50	164	231	0.3734(0.05380)	0.3468(0.05499)	<0.0001	<0.0001	Y
TP, g/L	65.0–85.0	162	227	60.95(6.447)	57.14(5.999)	<0.0001	<0.0001	Y
ALB, g/L	40.0–55.0	162	228	35.46(5.954)	31.44(4.833)	<0.0001	<0.0001	Y
Cellular immunological markers								
Lymphocyte, ×10^9^/L	1.2–3.8	164	231	1.100(0.5620)	0.8531(0.4362)	<0.0001	<0.0001	Y
CD3^+^T, ×10^6^/L	723–2,271	33	41	671.8(294.0)	494.4(334.5)	0.0747	0.1501	N
CD3^+^CD4^+^T, ×10^6^/L	396–1,309	33	40	408.0(186.9)	278.3(209.1)	0.1550	0.2377	N
CD3^+^CD8^+^T, ×10^6^/L	224–1,014	33	40	278.1(188.0)	196.7(158.9)	0.0893	0.1647	N
B, ×10^6^/L	118–645	33	39	93.40(69.73)	77.49(68.34)	0.4707	0.6015	N
NK, ×10^6^/L	61–607	32	39	126.8(89.12)	149.9(111.2)	0.6443	0.6989	N
CD3^+^T %	61.1–77	33	41	72.3 (13.99)	67.13 (13)	0.1050	0.1789	N
CD3^+^CD4^+^T %	25.8–41.6	33	40	40.75 (12.43)	39.01 (12)	0.5449	0.6596	N
CD3^+^CD8^+^T %	18.1–29.6	33	40	28.76 (9.875)	25.88 (11.73)	0.2672	0.3840	N
B %	9.02–14.1	33	39	9.592 (6.377)	10.49 (7.582)	0.5911	0.6798	N
NK %	10.04–19.78	32	39	16.86 (12.48)	19.57 (10.77)	0.3280	0.4557	N

Results are expressed as mean ± standard deviation (SD); Normality was assessed with the Shapiro–Wilk test; Student’s *t*-test was used when both groups satisfied normality, and the Mann–Whitney *U* test otherwise. *P* and FDR *q* values are shown; *q* < 0.05 was considered significant. Benjamini–Hochberg FDR correction was applied across all 45 markers within this comparison.WBC, White blood cell count; NLR: Neutrophil/Lymphocyte; hs-CRP, Hypersensitive C-reactive protein; SAA, serum amyloid A; IL-6, Interleukin-6; PCT, procalcitonin; PT, prothrombin time; APTT, activated partial thromboplastin time; TT, thrombin time; FIB, fibrinogen; FDP, fibrinogen degradation products; CK, creatine kinase; CK-MB, creatine kinase-MB; LDH, lactate dehydrogenase; HBDH, α-hydroxybutyrate dehydrogenase; IMA, ischemia-modified albumin; NT-proBNP, N-terminal pro–brain natriuretic peptide; hs-TnT, hypersensitive cardiac troponin T; DBIL, direct bilirubin; AST, aspartate aminotransferase; m-AST, mitochondrial aspartate aminotransferase; ALT, alanine aminotransferase; UREA, urea; CREA, creatinine; CYSC, cystatin C; RBC, red blood cell count; HGB, hemoglobin; HCT, hematocrit; TP, total protein; ALB, albumin.

### 3.5 Correlation between sex, age, and host response biomarkers in COVID-19 patients

**Figure 3:**
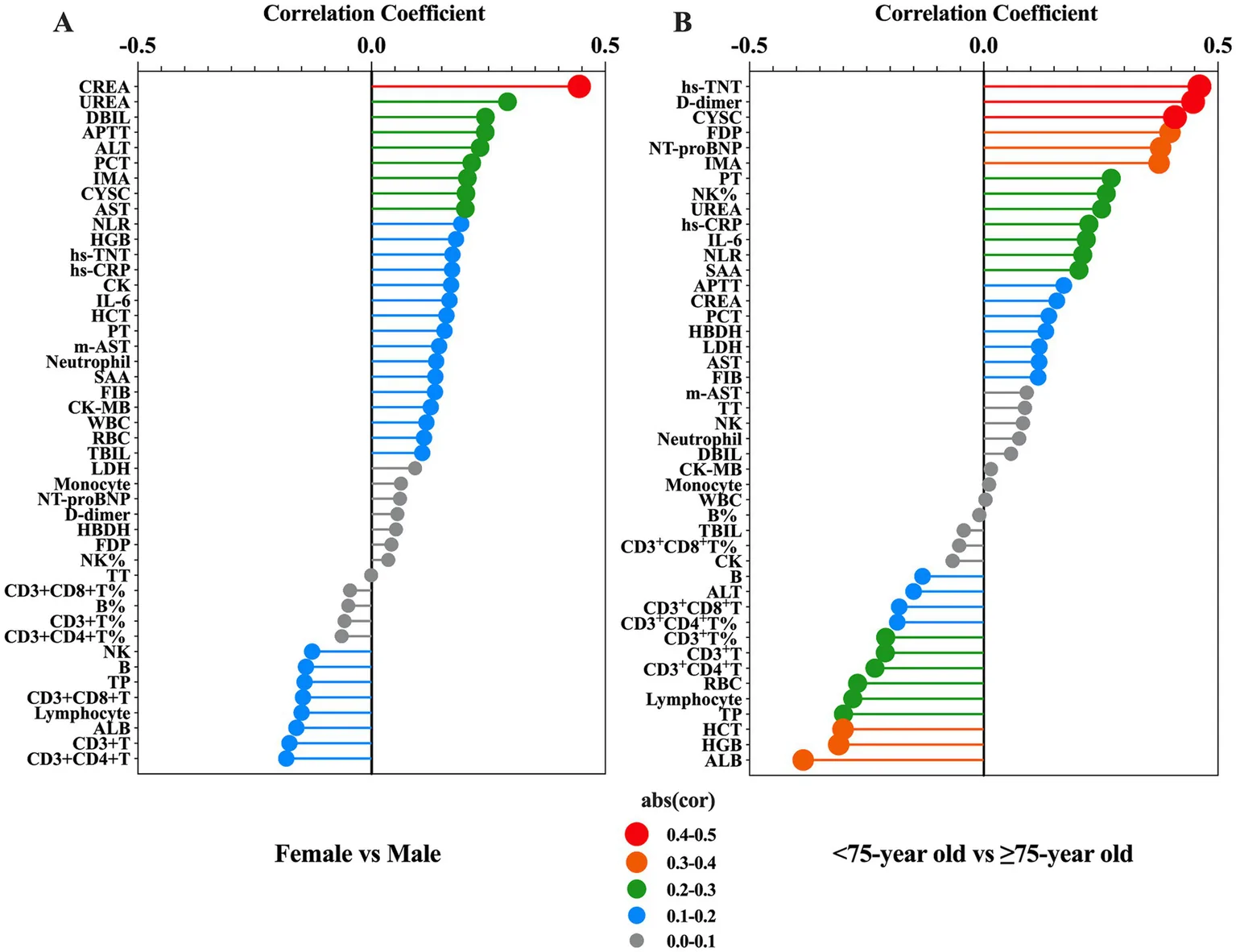
Spearman correlation analysis of **(A)** sex and **(B)** age with various host response biomarkers in COVID-19 patients. Spearman’s rank correlation was used to describe associations of sex and age with biomarker levels; these correlations were descriptive, were not adjusted for covariates, and were corrected for multiple testing with the same Benjamini–Hochberg false discovery rate correction across the 45 biomarkers.

We further explored the associations between sex, age, and host response biomarkers related to inflammatory responses, coagulation function, lymphocyte subsets, and cardiac, hepatic, and renal injury in COVID-19 patients. Spearman’s rank correlations were computed for sex and age against the 45 biomarkers, and statistical significance was defined by BH FDR *q* < 0.05 (Figure 3). As shown in Figure 3A, after FDR correction, 25 biomarkers correlated significantly with sex (male higher): WBC and neutrophil counts; inflammatory markers NLR, hs-CRP, PCT, and IL-6; coagulation parameters PT, APTT, and FIB; cardiac injury markers CK, IMA, and hs-TNT; hepatic injury markers DBIL, AST, m-AST, and ALT; renal injury biomarkers UREA, CREA, and CYSC; and RBC count, HGB, and HCT. In contrast, TP, ALB, and lymphocyte counts showed positive correlations (female higher). SAA, TT, D-dimer, FDP, CK-MB, LDH, HBDH, NT-proBNP, TBIL, and all T, B, and NK cell counts and percentages did not correlate significantly with sex after FDR correction. As shown in Figure 3B, after FDR correction, 24 biomarkers correlated significantly with age (positive rho, higher with increasing age): NLR, hs-CRP, PCT, IL-6, and SAA; coagulation parameters PT, APTT, FIB, D-dimer, and FDP; cardiac injury markers IMA, hs-TNT, and NT-proBNP; renal biomarkers UREA, CREA, and CYSC; and NK cell percentage. Age was negatively correlated with ALT, RBC count, HGB, HCT, TP, ALB, and lymphocyte counts.

## 4 Discussion

We investigated the associations between sex- and age-related differences and 45 biomarkers reflecting host response dysregulation following SARS-CoV-2 infection, encompassing key pathophysiological domains including immune response, inflammation, coagulation, and multi-organ dysfunction. Our findings confirm that males exhibit a higher proportion of severe COVID-19 cases than females, with disease severity predominantly affecting older adults, consistent with previous reports (2). Large-scale epidemiological studies have demonstrated that males have approximately 1.7-fold higher case fatality rates than females, and that advanced age is a major determinant of mortality (21, 22). Supporting this, national surveillance data indicate that the majority of COVID-19-related deaths occur in older adults, particularly those aged ≥65 years (23). In our cohort of 395 hospitalized patients, severe cases were disproportionately observed in males (76.19%) and in individuals aged ≥75 years (78.57%), further reinforcing the increased vulnerability of older males to severe disease. Collectively, these findings suggest that the higher incidence of severe COVID-19 among older males may be associated with biological differences related to age and sex, including differential virus-induced organ injury and variations in host immune responses.

COVID-19 is increasingly recognized as a systemic disease capable of affecting multiple organ systems, including the heart, lungs, brain, kidneys, pancreas, skeletal muscle, adipose tissue, nervous system, vascular endothelium, and coagulation pathways (24, 25). This widespread involvement may result from direct viral cytopathic effects such as cellular injury, tissue hypoxia, and the induction of prothrombotic and hypercoagulable states as well as from dysregulated immune activation and systemic inflammation. Among the underlying mechanisms, angiotensin-converting enzyme 2 (ACE2) plays a central role in COVID-19 associated multi-organ dysfunction. The spike (S) protein of SARS-CoV-2 binds to ACE2 on the host cell membrane and, together with transmembrane protease serine 2 (TMPRSS2), facilitates viral entry (26). The differential expression of ACE2 and TMPRSS2 across tissues including the lungs, kidneys, liver, and heart provides a molecular basis for multi-organ susceptibility (27–30).

ACE2 expression and activity are reported to be higher in males than in females and age-related upregulation of ACE2 and TMPRSS2 in myocardial cells has been described (3). Consistent with these reported mechanisms, our sex-stratified analyses revealed significantly higher coagulation markers (PT, APTT, FIB), myocardial injury biomarkers (CK, IMA, hs-TNT), liver injury markers (DBIL, AST, m-AST, ALT), and renal injury indicators (UREA, CREA, CYSC) in male versus female patients. Similarly, age-stratified analysis showed that older patients (≥75 years) exhibited significantly elevated biomarkers of coagulation dysfunction (PT, APTT, D-dimer, FIB, FDP), myocardial injury (IMA, hs-TNT, NT-proBNP), and renal impairment (UREA, CREA, CYSC). Estrogen-mediated modulation of ACE2 and age-related RAAS activation with increased angiotensin II levels represent plausible mechanisms underlying these observed differences, though direct measurements of these pathways were beyond the scope of this study (31–33). However, ACE2, TMPRSS2, and sex hormone levels were not assessed in this retrospective cohort; thus, the involvement of these potential pathways remains speculative, and the observed associations should be regarded as indirect evidence that warrants further validation in future mechanistic studies.

Regarding laboratory findings, most patients with severe COVID-19 in our cohort exhibited a hyperinflammatory state accompanied by lymphocytopenia, a hallmark of critical coronavirus infection previously described in SARS-CoV. In COVID-19 progression, dysregulated inflammatory responses play a central role, and excessive cytokine release is strongly associated with disease severity (34). Lymphocytopenia may result from apoptosis-related mechanisms, including upregulation of Fas and Fas ligand (FasL) in T, B, and natural killer (NK) cells. In addition, increased expression of programmed cell death protein 1 (PD-1) and T-cell immunoglobulin and mucin-domain containing-3 (TIM-3) on CD4^+^ and CD8^+^ T cells promotes T-cell exhaustion and aberrant cytokine production, thereby aggravating immune dysfunction (35).

Sex- and age-related immune heterogeneity further influences disease outcomes. Female patients generally experience milder clinical manifestations and lower mortality, potentially owing to stronger innate and adaptive immune responses (36). Multiple immune cell subsets express estrogen receptors, suggesting that estrogen-mediated signaling enhances antiviral immunity (37). However, this immunological advantage declines with age (38). Consistent with prior studies (39, 40), male patients in our cohort exhibited heightened inflammatory responses, reflected by elevated WBC and neutrophil counts, NLR, CRP, PCT, and IL-6, and reduced lymphocyte counts. Absolute counts and percentages of CD3^+^, CD4^+^, CD8^+^, B, and NK cells did not differ significantly between sexes, indicating that overall lymphopenia, rather than preferential depletion of a specific T-cell lineage, characterized male patients. Aging further amplifies immune dysregulation. Advanced age is associated with increased expression of inflammatory, senescence-associated, and coronavirus susceptibility genes within specific immune cell subsets (41). SARS-CoV-2 infection may intensify age-related immune polarization and inflammatory pathway activation. Enhanced viral susceptibility gene expression, accumulation of pro-inflammatory mast cells, and T-cell depletion collectively promote cytokine storm and lymphopenia (13, 42), thereby increasing vulnerability in older adults. In agreement with this mechanism, older patients in our study exhibited elevated NLR, hs-CRP, IL-6, and SAA levels and reduced lymphocyte counts; T-cell subset counts and percentages did not differ significantly after FDR correction, and reduced TP and ALB may also reflect chronic comorbidity and nutritional status.

This study has several limitations. First, as a single-center retrospective study with a moderate sample size, the findings may not be generalizable to other populations or healthcare settings. Second, data on vaccination status and SARS-CoV-2 variant types were not available, both of which are important confounders during the Omicron-predominant period of this study. Third, the exclusion of patients with chronic inflammatory conditions, autoimmune diseases, and malignancies may have selected a healthier cohort, potentially underestimating the full impact of comorbidities on COVID-19 severity. Fourth, our age cutoff of 75 years, while clinically meaningful, may limit comparability with studies using lower thresholds. Fifth, all laboratory measurements were obtained within 48 h of diagnosis and represent a single time point; moreover, the interval from symptom onset to sampling was not recorded directly, and symptom-onset-to-discharge duration differed between severity groups. We adjusted for onset-to-discharge duration in multivariable models, but residual time-course bias cannot be excluded. Sixth, treatment data, including corticosteroids and other immunomodulatory therapies administered before blood sampling, were not available, and such treatments can influence leukocyte and lymphocyte counts. Seventh, although multivariable adjustment and co-infection-restricted sensitivity analyses were performed, residual confounding by pre-existing comorbidity and organ dysfunction cannot be excluded. Finally, ACE2, TMPRSS2, and sex hormone levels were not measured, and the diagnostic performance of individual biomarkers for predicting severity was not evaluated via receiver operating characteristic analysis, which warrants future investigation. Despite these limitations, the comprehensive profiling of 45 biomarkers across multiple organ systems in a well-characterized cohort provides valuable insights into the associations between sex- and age-related disparities in COVID-19 outcomes.

In conclusion, sex and age differences were associated with hospitalization rates and the severity of COVID-19, as well as with distinct biomarker patterns of inflammation, coagulation, immune dysregulation, and multi-organ injury. Because this was an observational study, these associations should not be interpreted as causal. Sex- and age-related factors may merit consideration in future mechanistic studies and risk-stratification research, but therapeutic implications require prospective evaluation.

## Data Availability

The original contributions presented in the study are included in the article/supplementary material, further inquiries can be directed to the corresponding authors.
